# Early-life stress and inflammation: A systematic review of a key experimental approach in rodents

**DOI:** 10.1177/2398212820978049

**Published:** 2020-12-28

**Authors:** Ethan G. Dutcher, E.A. Claudia Pama, Mary-Ellen Lynall, Shahid Khan, Menna R. Clatworthy, Trevor W. Robbins, Edward T. Bullmore, Jeffrey W. Dalley

**Affiliations:** 1 Department of Psychology, University of Cambridge, Cambridge, UK; 2 Department of Psychiatry, University of Cambridge, Cambridge, UK; 3 Molecular Immunity Unit, MRC Laboratory of Molecular Biology, Cambridge, UK; 4 GlaxoSmithKline Research & Development, Stevenage, UK

**Keywords:** Maternal separation, early-life adversity, depression, chronic stress, cytokines, immune system, neuroimmune responsiveness

## Abstract

Repeated maternal separation is the most widely used pre-clinical approach to investigate the relationship between early-life chronic stress and its neuropsychiatric and physical consequences. In this systematic review, we identified 46 studies that conducted repeated maternal separation or single-episode maternal separation and reported measurements of interleukin-1b, interleukin-6, interleukin-10, tumour necrosis factor-alpha, or microglia activation and density. We report that in the short-term and in the context of later-life stress, repeated maternal separation has pro-inflammatory immune consequences in diverse tissues. Repeated maternal separation animals exhibit greater microglial activation and elevated pro-inflammatory cytokine signalling in key brain regions implicated in human psychiatric disorders. Notably, repeated maternal separation generally has no long-term effect on cytokine expression in any tissue in the absence of later-life stress. These observations suggest that the elevated inflammatory signalling that has been reported in humans with a history of early-life stress may be the joint consequence of ongoing stressor exposure together with potentiated neural and/or immune responsiveness to stressors. Finally, our findings provide detailed guidance for future studies interrogating the causal roles of early-life stress and inflammation in disorders such as major depression.

## Introduction

Early-life stress (ELS), synonymous in the human literature with childhood maltreatment (Danese et al., 2007; Hodel et al., 2015), is associated with many adverse neuropsychiatric and physical health outcomes later in life. ELS has repeatedly been associated with increased risk for later-life diagnosis of depressive disorders including major depressive disorder and dysthymia, anxiety disorders including post-traumatic stress disorder (PTSD), social phobia, generalised anxiety disorder, and panic disorder, and substance use disorders such as alcohol use disorder (Edwards et al., 2003; Gibb et al., 2007; Spinhoven et al., 2010; Teicher and Samson, 2013; Wright et al., 2009). At least two meta-analyses have demonstrated strong associations between child abuse and adverse physical health outcomes in adulthood, with child abuse being associated especially with increased risk of neurological, musculoskeletal, respiratory, cardiovascular, and gastrointestinal symptoms and conditions (Irish et al., 2010; Wegman and Stetler, 2009). In addition to likely increasing the risk of developing these disorders, ELS also appears to predispose to a more severe clinical course of at least some of them. A large meta-analysis concluded that among patients who suffer from depressive disorders, a history of ELS is associated with an increased number of depressive episodes, increased duration of the current depressive episode, and decreased responsiveness to treatment (Nanni et al., 2012). Similarly, a recent meta-analysis of bipolar disorder patients concluded that a history of childhood maltreatment is associated with earlier disorder onset, increased severity and number of depressive and manic episodes, and increased risk of suicide attempts, anxiety disorders including PTSD, substance use disorders, and rapid cycling between mania and depression (Agnew-Blais and Danese, 2016).

Many studies have also demonstrated that people with a history of ELS have higher inflammatory responses to acute stress and higher peripheral pro-inflammatory signalling in general. For example, a history of ELS has been associated with a larger increase in circulating interleukin (IL)-6 when people are asked to deliver a speech about their job qualifications and perform mental arithmetic in front of expressionless judges of their performance (Carpenter et al., 2010; Janusek et al., 2017; Pace et al., 2006). In addition, a recent meta-analysis showed that people with a history of childhood trauma have significantly elevated circulating IL-6, tumour necrosis factor-alpha (TNF-α), and C-reactive protein (CRP), in the absence of any specific laboratory stressor, although not necessarily in the absence of the stresses of everyday adult life (Baumeister et al., 2016). What is not clear, however, is whether this propensity to higher inflammation in later life plays a causal role in the neuropsychiatric or physical health consequences of ELS. Such questions of causality are well-suited for investigation using animal models because elements of the immune system may be selectively targeted using pharmacological, genetic, or cellular interventions during ELS, adult stress, or both. Research in experimental animals holds the additional advantage compared to work in humans of allowing access to brain tissue at any point during and after ELS, enabling precise characterization of its molecular and cellular consequences in the central nervous system (CNS).

**Figure 1.**
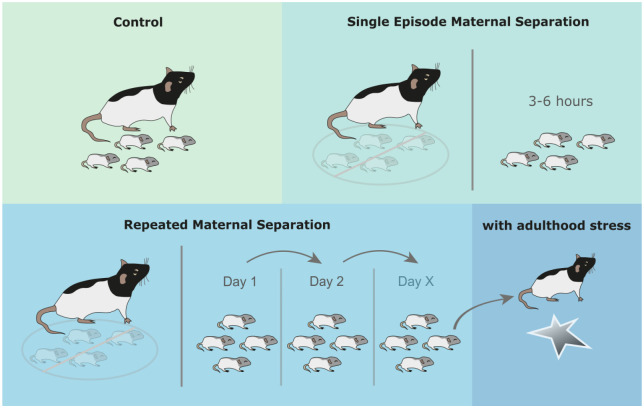
The maternal separation (MS) procedure. A single episode of maternal separation (SEMS) involves separating rat or mouse pups from their dam, most commonly for 3 h. During this period, the physical and emotional needs of pups go unmet by their mother, resembling neglect. In repeated maternal separation (RMS), this procedure is repeated daily, most commonly commencing on post-natal day (PND) 1 or 2 and concluding on either PND 14 or 21. Other aspects of the protocol are less consistent across studies, such as whether pups are also separated from one another, whether pups are warmed or not warmed during separation, whether separation occurs during the light or dark cycle, and whether control animals are handled or not handled during early life.

The most widely used animal model for investigating the relationships between ELS and its psychiatric and physical consequences is repeated maternal separation (RMS) (Andersen, 2015; Schmidt et al., 2011). This procedure involves repeatedly separating rat or mouse pups from their mother, most commonly for 3–6 h each day, beginning on either post-natal day (PND) 1 or 2, and continuing through either PND 14 or 21. During these separation periods, around 35%–40% of studies also separate pups from one another; this combined separation is sometimes referred to as early deprivation (Pryce and Feldon, 2003). More information on RMS can be found in Figure 1.

While many studies have examined the effects of RMS on the immune system, the results are somewhat inconsistent. For example, within the same tissues, there are many reports of increased, decreased, or unaltered expression of specific cytokines (Kruschinski et al., 2008; Roque et al., 2016; Wang et al., 2017). Furthermore, there are many reports suggesting that RMS causes long-lasting effects on a diverse array of not just immunological outcomes but also depressive-like behaviour, anxiety-like behaviour, and gastrointestinal tract function, among others (Dallé et al., 2017; Mizoguchi et al., 2019; Oines et al., 2012). Equally, however, for some of these outcomes, there are many reports that find no long-lasting effect (Bassey and Gondré-Lewis, 2019; Harrison et al., 2014; Stuart et al., 2019).

In this systematic review, we sought to establish a clearer picture of the effects of RMS on the immune system. We hypothesised that much of the variability in the literature could be explained by two variables: (1) whether assessments were made shortly after the conclusion of RMS or instead closer to or during adulthood and (2) whether the animals experienced any further stress following RMS. While a wide variety of immunological parameters have been measured in RMS animals, we focused our review on a manageable subset of these intended to provide a representative picture of clinically significant pro-inflammatory innate immune system changes in the periphery and CNS. Specifically, we included measurements in any tissue of the four cytokines most commonly assayed in RMS studies (IL-1β, IL-6, IL-10, and TNF-α), as well as interpretable measures of effects on microglia, the resident immune cells of the CNS (Graeber, 2010), including the density of microglia in brain tissue and their degree of activation. Included cytokines are all pro-inflammatory except IL-10, which has predominantly anti-inflammatory actions (Pestka et al., 2004; Sabat et al., 2010).

## Methods

The full methods can be found in the Supplemental material. In brief, a search was conducted, results were screened against eligibility criteria, and then included findings were presented descriptively and summarised in the form of most frequent outcomes. Measurements were considered to assess long-term effects of RMS if they were collected more than 3 weeks after its conclusion, and short-term effects otherwise. Reported measurements of cytokine expression in blood always refer to protein level. For non-blood tissues, because protein and mRNA results were generally concordant (Supplemental Figure S1, and see Avitsur et al., 2006; Ganguly et al., 2019), the assay substrate may not be specified in the main text but can be found in Supplemental Figure S2, along with the species and gender of the animals in each included study.

## Results

### Overview

**Figure 2.**
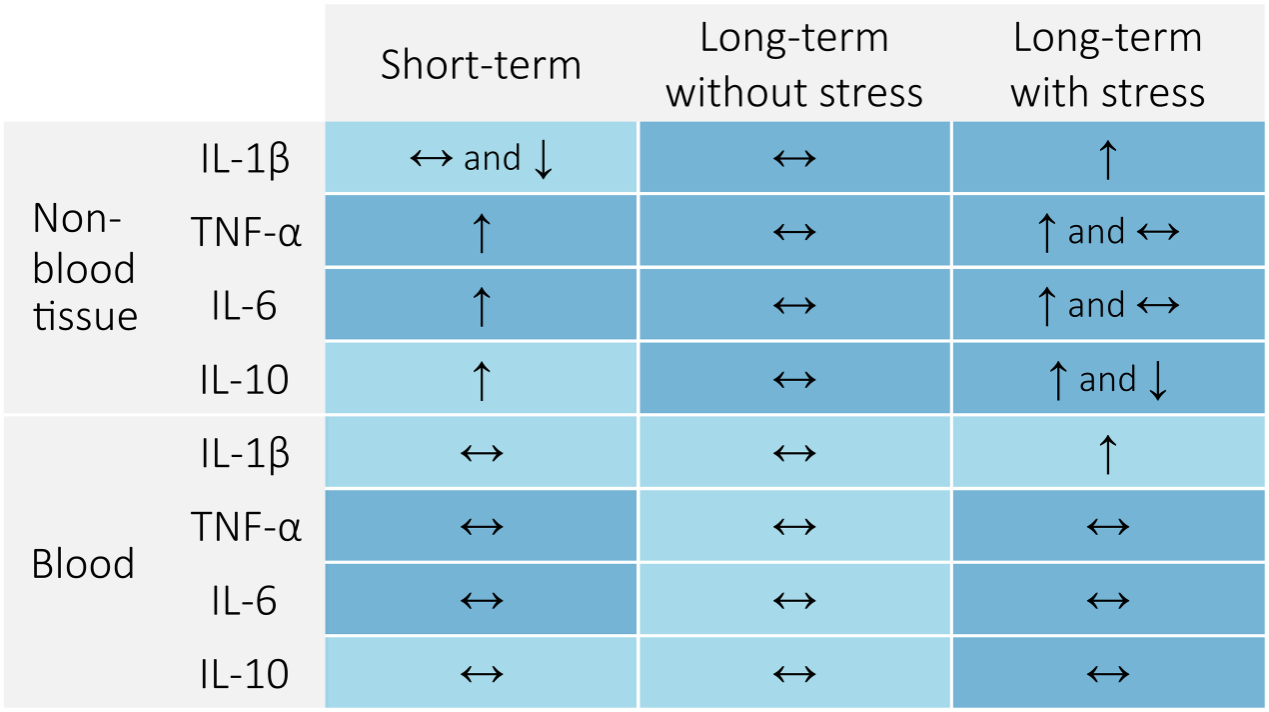
Effects of repeated maternal separation (RMS) on cytokine expression. The most commonly reported outcomes (increase, decrease, or no change) are summarised for each cytokine for each stress condition, in both blood and non-blood tissue. Dark blue shading indicates a high level of confidence (three or more studies supporting each outcome), whereas light blue shading indicates low confidence. Measurements were considered short-term if tissue samples were collected within 3 weeks after the conclusion of RMS, and long-term otherwise.

The most frequently reported effects of RMS on cytokine expression were determined and are displayed in Figure 2. In the short-term, RMS generally increases TNF-α, IL-6, and IL-10 in non-blood tissue while leaving these unaffected in blood (plasma, serum, or the supernatant of cultured whole blood). Without further stress, RMS has no long-term effect on cytokine expression in both non-blood tissues and blood. However, if further stress is applied, RMS animals exhibit increased IL-1β, TNF-α, IL-6, and IL-10 in non-blood tissue, although studies also regularly reported no change in TNF-α or IL-6, and a decrease in IL-10. In both contexts involving recent stress, increases in cytokine expression were much more commonly observed in non-blood tissue than in blood. The effects of RMS on microglia are described in the subsequent sections and summarised in Supplemental Figure S3.

### Short-term effects of maternal separation

#### IL-1β

Several studies have examined the short-term effects of maternal separation (MS) on hippocampal IL-1β. In animals sacrificed immediately after the final MS episode, RMS increased hippocampal IL-1β mRNA levels by roughly 20 times compared to never-stressed animals (Roque et al., 2016). When sacrificed 24 h after the final episode, IL-1β expression was still significantly elevated, but reduced to 2–2.5 times the level of unstressed animals (Roque et al., 2016). In animals given intraperitoneal (IP) saline injections immediately after their final RMS episode and sacrificed 90 min later, hippocampal IL-1β protein showed a trend increase in RMS animals (Saavedra et al., 2017); here, because both RMS and control animals received IP injections, both groups experienced a brief stressor which may have reduced the statistical power to detect an RMS-induced effect on cytokine expression. A further study using a shorter RMS protocol reported no effect of RMS on hippocampal IL-1β protein in animals sacrificed on the final day of RMS (Giridharan et al., 2019). Animals sacrificed immediately after single-episode maternal separation (SEMS) were not found to have increased hippocampal IL-1β expression relative to unstressed animals (Roque et al., 2016). However, among animals sacrificed immediately at the conclusion of an episode of MS, those who had undergone RMS had roughly five times the hippocampal IL-1β level that SEMS animals had (Roque et al., 2016). Altogether these findings suggest that RMS may cause hippocampal IL-1β to undergo daily cycling with peaks shortly after each MS episode, with a rapid return towards normal until the stress is applied again. The daily peak appears to rise with each additional repetition such that eventually the daily elevation does not normalise even by the start of the next day’s episode.

Findings in other regions of the brain suggest that the effects of RMS on brain IL-1β are probably region-specific, while SEMS likely has no effect on brain IL-1β in any region. Two studies reported no effect of RMS, in the hypothalamus, in the same study that demonstrated a profound increase in hippocampal IL-1β immediately after the final episode (Roque et al., 2016), and in the prefrontal cortex (PFC), in animals sacrificed on the day of the final RMS episode (Giridharan et al., 2019). Two studies reported no effect of SEMS on hypothalamic IL-1β (Roque et al., 2016; Zajdel et al., 2019), mirroring the lack of effect in the hippocampus. Two final studies reported a decrease in IL-1β expression in RMS animals, specifically in the prelimbic PFC at almost 3 weeks following the final RMS episode (Majcher-Maślanka et al., 2019), and in a whole-brain homogenate at 48 h after the final episode (Dimatelis et al., 2012). Only one included study looked at the short-term effects of MS on IL-1β in a non-blood tissue other than the brain, finding no effect of SEMS on liver IL-1β (Zajdel et al., 2019).

The effects of RMS on plasma IL-1β appear short-lived. Two studies found no effect of RMS on plasma IL-1β, at 5 and 15 days following the final RMS episode (Grassi-Oliveira et al., 2016), and in animals given an IP saline injection shortly after the final episode and sacrificed 90 min later (Saavedra et al., 2017). However, another study reported that RMS decreased plasma IL-1β both immediately after the final RMS episode and 24 h later, to roughly 65%–70% and 80%–85% of the level of unstressed animals, respectively (Roque et al., 2016). In contrast to RMS, SEMS did not elicit this decrease in animals sacrificed immediately after the episode, but rather appeared to increase IL-1β plasma levels compared with unstressed animals (Roque et al., 2016).

#### TNF-α

Two included studies examined the short-term effects of MS in the hippocampus. Roque et al. (2016) found in animals sacrificed immediately after MS that while SEMS caused a modest increase in hippocampal TNF-α expression, the mean hippocampal TNF-α level in RMS animals sacrificed at the same time was indistinguishable from that of never-stressed animals. This suggests that with repeated episodes of MS, the ability of each daily episode to raise TNF-α expression may decrease, which is the opposite of the finding regarding hippocampal IL-1β expression. A significant increase in hippocampal TNF-α was found in RMS animals 24 h following separation compared with animals that had never been stressed, although the magnitude of this difference was small. Corroborating this finding, increased hippocampal TNF-α was reported in rats sacrificed on the final day of RMS (Giridharan et al., 2019). Altogether, these results suggest that RMS likely increases hippocampal TNF-α, but that this effect may be modest compared to the increase in IL-1β. Furthermore, the peak daily hippocampal TNF-α level may decrease rather than increase with repeated exposure.

In other brain regions, one study reported increased TNF-α expression in the PFC in animals sacrificed on the day of their final RMS episode (Giridharan et al., 2019), and another found that RMS animals sacrificed immediately after their final episode had higher hypothalamic TNF-α than SEMS animals sacrificed at the same time (Roque et al., 2016). However, this latter increase in RMS animals was not significant when compared either to animals that were never stressed or to animals that were sacrificed at 24 h following their final RMS episode. In addition, there was no suggestion of a difference in hypothalamic TNF-α between RMS animals sacrificed 24 h after their final episode and never-stressed animals. These findings suggest that RMS may cause modest increases in TNF-α expression in the hypothalamus and PFC, but, contrary to the findings in the hippocampus, that these increases may be greater at the conclusion of RMS rather than 24 h later, and that peak expression may increase with chronicity.

Most studies to date report no short-term effect of MS on TNF-α expression in non-brain tissues. Studies have found no effect of RMS or SEMS on plasma TNF-α (Barouei et al., 2015; Roque et al., 2016; Saavedra et al., 2017) or of SEMS on liver TNF-α (Zajdel et al., 2019). In colon tissue, RMS during PND 5-9 increased TNF-α mRNA at sacrifice immediately after the final episode, although RMS but not control animals received daily IP saline injections (Li et al., 2017).

#### IL-6

Included studies generally suggest that while RMS can increase IL-6 expression in blood, this increase is likely short-lived. Roque et al. (2016) demonstrated that both RMS and SEMS cause similar increases in plasma IL-6 at the immediate conclusion of an MS episode. This study also demonstrated that by 24 h following the conclusion of an RMS episode, plasma IL-6 had decreased to below the level of unstressed animals. If IL-6 does indeed rapidly change from elevated to decreased within a 24-h period following MS, these changes may be difficult to detect. Indeed, three studies found no effect of RMS on plasma IL-6, in animals sacrificed on the final day of RMS (Moya-Pérez et al., 2017; Saavedra et al., 2017) or 10 days later (Barouei et al., 2015).

Findings regarding the effect of MS on IL-6 in tissues other than blood generally seem to suggest that RMS but not SEMS increases IL-6 expression in a variety of tissues, at least for a short period of time. In the hypothalamus, among animals sacrificed immediately following an episode of MS, one study demonstrated that RMS animals had elevated hypothalamic IL-6 mRNA compared to unstressed animals, while SEMS animals did not (Roque et al., 2016). Again, hypothalamic IL-6 returned to baseline within 24 h following RMS (Roque et al., 2016). Another study confirmed the lack of an effect of SEMS on not only hypothalamic IL-6 but also liver IL-6, even in animals sacrificed immediately following the episode (Zajdel et al., 2019). RMS has also been reported to increase IL-6 in the PFC on the final day of RMS (Giridharan et al., 2019) and in colon tissue within 3 days after RMS (Li et al., 2017; O’Malley et al., 2011). The only non-blood tissue in which IL-6 has been reported to be unaffected by RMS is the hippocampus, even in animals sacrificed immediately following the final episode (Giridharan et al., 2019; Roque et al., 2016).

#### IL-10

Studies investigating the short-term effects of MS on IL-10 expression have generally found either an increase or no change in RMS animals. In animals sacrificed on the final day of RMS, increases in IL-10 expression were found in PFC and small intestine but not in hippocampus or serum (Giridharan et al., 2019; Moya-Pérez et al., 2017). A different study collected plasma from both males and females at 5, 15, and 35 days following RMS, and in almost all cases no effect on IL-10 was reported, with the exception of an isolated finding in males at 15 days following RMS of increased IL-10 (Grassi-Oliveira et al., 2016). Finally, one study found a decrease in IL-10 in RMS animals in whole-brain homogenates (Dimatelis et al., 2012).

#### Microglial activation and density

Included studies consistently report that RMS leads to microglial activation. Three studies measured microglial activation within 3 days after the conclusion of RMS, and all three demonstrated increased microglial activation on morphological analysis, regardless of whether they used a binary classification system (Roque et al., 2016; Saavedra et al., 2017) or directly assessed soma area and arborization area (Baldy et al., 2018). This finding was consistent across both CNS regions examined: in the hippocampus, specifically the hilus (Roque et al., 2016; Saavedra et al., 2017) and CA3 (Saavedra et al., 2017), and in the medulla (Baldy et al., 2018). With respect to microglia density, there is no consensus regarding the short-term effects of RMS. Some studies report decreased microglia density, including in CA3 and the hippocampal hilus (Saavedra et al., 2017) and the prelimbic PFC (Majcher-Maślanka et al., 2019), while others reported no effect in the hippocampal hilus (Roque et al., 2016) and increased microglia density in the medulla (Baldy et al., 2018).

### Long-term effects of RMS in the absence of later-life stress

The majority of studies have found no long-term effect of RMS on IL-1β expression, in plasma (Kruschinski et al., 2008), lung (Avitsur et al., 2006; Kruschinski et al., 2008), colon (Lennon et al., 2013), spinal cord (Genty et al., 2018), or hippocampus (Zhu et al., 2017). Only Barreau et al. (2004) reported increased IL-1β in adult RMS animals not subjected to further stress, in the colon, liver, and spleen.

In the absence of later-life stress, RMS does not have significant effects on TNF-α expression in a range of regions and tissues, including hippocampus (Banqueri et al., 2019; Zhu et al., 2017), dorsal striatum (Banqueri et al., 2019), PFC (Banqueri et al., 2019), lung (Avitsur et al., 2006), colon (Lennon et al., 2013; Pierce et al., 2014), genitourinary tract (Pierce et al., 2014), splenocytes (Kiank et al., 2009), or plasma (Barouei et al., 2015; Grassi-Oliveira et al., 2016). However, Riba et al. (2017, 2018) reported elevated levels of TNF-α in the small intestine of RMS animals sacrificed at PND 50. The reasons for this discrepancy are unclear but may include use of pair housing and sacrifice closer to the conclusion of RMS than most other included studies of long-term effects. The reduced opportunity for social play may have slowed the normalisation of the stress response and consequent immune effects in these animals, such that it did not occur by the relatively early time of sacrifice (Brenes Sáenz et al., 2006; Hui et al., 2011; Odeon and Acosta, 2019; Veena et al., 2009).

In general, RMS has no lasting impact on IL-6 in a variety of brain regions and tissues, including the hippocampus (Zhu et al., 2017), dorsal striatum (Banqueri et al., 2019), PFC (Banqueri et al., 2019), plasma (Barouei et al., 2015), spinal cord (Genty et al., 2018), colon (Fuentes et al., 2016; Pierce et al., 2014), genitourinary tract (Pierce et al., 2014), and lung (Avitsur et al., 2006). Nevertheless, increased IL-6 mRNA has been reported in the hippocampus (Banqueri et al., 2019) and colon (Lennon et al., 2013).

In a similar vein, RMS alone appears to have no lasting effects on IL-10 expression in plasma (Grassi-Oliveira et al., 2016), splenocyte culture with lipopolysaccharide (LPS) (Kiank et al., 2009), colon, and genitourinary tract (Pierce et al., 2014, 2016). As with IL-1β, Barreau et al. (2004) alone reported increased IL-10, in the colon, liver, and spleen.

To date, only one study has looked at microglial activation in adult animals not subjected to further stress (Ganguly et al., 2018). Here, no effects were found in relation to microglial soma area, summed microglial process length, or microglial process end-point count in the prelimbic PFC (Ganguly et al., 2018). In terms of microglial density, while one study reported an increase in CA3, dorsal striatum, and nucleus accumbens (Banqueri et al., 2019), another study found no effect in the prelimbic PFC (Ganguly et al., 2018).

### Long-term effects of RMS in the presence of later-life stress

Studies measuring the effects of RMS on IL-1β expression in animals subjected to further stress commonly report increased IL-1β expression in RMS animals, including in the hippocampus (Amini-Khoei et al., 2017, 2019; Wang et al., 2017; Zhu et al., 2017), PFC (Wang et al., 2017), paraventricular nucleus (PVN) (Tang et al., 2017), striatum (Dallé et al., 2017), cerebrospinal fluid (Réus et al., 2013), colon (Amini-Khoei et al., 2019), kidney (De Miguel et al., 2018), lung (Avitsur et al., 2006), and serum (Réus et al., 2013; Wang et al., 2017). However, several studies report no change or even decreased IL-1β expression in RMS animals, although often in the context of a direct inflammatory insult, possibly suggesting a degree of psychosocial stress-evoked immune cell habituation or exhaustion. While no effect of RMS was found in serum (Avitsur et al., 2013; Breivik et al., 2015) or spleen (De Miguel et al., 2018), among animals subjected first to mild psychosocial stress and then shortly after to high-dose LPS, those who had undergone RMS had decreased serum IL-1β (Avitsur et al., 2013). In another case involving both psychological distress and a severe physical inflammatory insult, nerve compression trauma increased spinal cord IL-1β mRNA in control but not RMS animals (Genty et al., 2018). The experimental methodologies of the later-life stressors are summarised in Supplemental Figure S2.

Most studies in which animals underwent a second stress have reported an increase in TNF-α in RMS animals, although generally in non-blood tissues rather than blood. Regarding just the brain, an increase in TNF-α expression in RMS animals was reported in the hippocampus (Amini-Khoei et al., 2017; Han et al., 2019; Pinheiro et al., 2015; Zhu et al., 2017), although in Han et al. (2019), this difference was not tested statistically; the PFC (Ganguly et al., 2019; Pinheiro et al., 2015), although only in males but not females in Ganguly et al. (2019); the PVN (Tang et al., 2017); the striatum (Dallé et al., 2017); and the nucleus accumbens in males but not females (Ganguly et al., 2019). Several studies, however, did not find any effect of RMS in the hippocampus (Viola et al., 2019; Wang et al., 2017) and PFC (Amini-Khoei et al., 2019; Viola et al., 2019). Reports measuring TNF-α expression in other non-blood tissues generally find either increased expression or no change in RMS animals. While increases have been reported in large intestine tissue (Amini-Khoei et al., 2019), the reproductive tract (Pierce et al., 2014), and lung (Avitsur et al., 2006), a lack of effect has been reported in the bladder or colon (Pierce et al., 2014), lung (Kruschinski et al., 2008), spleen (De Miguel et al., 2018), and splenocyte culture with LPS (Kiank et al., 2009). In most studies that have looked at TNF-α in blood in animals exposed to later-life stress, no effect of RMS has been shown, including in the serum (Avitsur et al., 2013; Carboni et al., 2010; Wang et al., 2017), plasma (Barouei et al., 2015), and whole blood, with or without ex vivo stimulation with LPS (Desbonnet et al., 2010; O’Mahony et al., 2009) or concanavalin A (Desbonnet et al., 2010). However, a few studies have found a long-term effect of RMS on blood TNF-α, with two studies finding an increase (Do Prado et al., 2016; Réus et al., 2013), and one finding a decrease in females but not males, and only after low-dose but not high-dose LPS or saline administration (Avitsur et al., 2013).

In animals subjected to later-life stress, in contrast to the relatively consistent findings of increased IL-1β and TNF-α in RMS animals, especially in non-blood tissue, the findings regarding IL-6 are mixed. Many studies have found no effect of RMS on IL-6, including in lung tissue or bronchoalveolar lavage fluid (Kruschinski et al., 2008; Vig et al., 2010), the reproductive tract (Pierce et al., 2014), the bladder (Pierce et al., 2016), the colon (Fuentes et al., 2016; Pierce et al., 2014), the kidney and spleen (De Miguel et al., 2018), the hypothalamic PVN (Tang et al., 2017), the medial PFC (Viola et al., 2019), the spinal cord (Genty et al., 2018), plasma (Barouei et al., 2015; Kruschinski et al., 2008), serum (Avitsur et al., 2013; Carboni et al., 2010), and whole blood cultured ex vivo with LPS (Desbonnet et al., 2010; O’Mahony et al., 2009). However, there are a number of studies that have reported an increase in IL-6 in RMS animals, specifically in the colon and bladder (Pierce et al., 2016), lung (Avitsur et al., 2006), whole blood cultured with concanavalin A but not saline (Desbonnet et al., 2010), striatum (Dallé et al., 2017), and hippocampus (Han et al., 2019; Zhu et al., 2017), albeit not tested statistically in Han et al. (2019). Finally, one study reported decreased IL-6 expression in RMS animals, specifically in the medial PFC (Viola et al., 2019).

As with IL-6, findings regarding IL-10 expression in animals exposed to a second stress are mixed. Consistent with a pro-inflammatory state, five studies have reported decreased IL-10 expression in RMS animals, in the striatum, concurrently with increased IL-1β, IL-6, and TNF-α (Dallé et al., 2017), the ventral hippocampus, together with higher IL-1β (Wang et al., 2017), splenocytes cultured with LPS, along with non-significantly elevated TNF-α (Kiank et al., 2009), the serum, together with increased IL-1β and TNF-α (Réus et al., 2013), and colon tissue, alongside increased interferon gamma (Shao et al., 2019). However, most studies have reported no effect of RMS on IL-10 expression, including in whole blood cultured with or without LPS or concanavalin A (Desbonnet et al., 2010; O’Mahony et al., 2009), serum (Breivik et al., 2015; Carboni et al., 2010; Wang et al., 2017), plasma (Kruschinski et al., 2008), PFC (Pinheiro et al., 2015; Wang et al., 2017), colon (Pierce et al., 2014, 2016), bladder (Pierce et al., 2016), and lung (Kruschinski et al., 2008), and an increase in IL-10 has been reported by three studies, in the genitourinary tract (Pierce et al., 2014), hippocampus (Pinheiro et al., 2015), and bladder (Pierce et al., 2016).

Only two included studies measured microglia activation or density in RMS animals in the context of later-life stress. In animals that received daily IP saline injections from PND 28 to 42, at 2 weeks after the conclusion of injections, RMS animals had slightly but significantly reduced hippocampal microglial process length and count, consistent with a more activated phenotype on average (Han et al., 2019). Among animals subjected to an additional 2 weeks of daily 2-h restraint stress from PND 42 to 56, the same effects of MS on process length and number described above were observed, but here the effects were robust enough to also be detected using a morphological classification-based approach. Regarding microglia density, one study found that MS confers a vulnerability lasting at least into young adulthood to a greater increase in spinal cord microglia in response to compression trauma of a nearby nerve (Mizoguchi et al., 2019).

## Discussion

In this systematic review, we sought to establish a clearer picture of the effects of ELS on the innate immune system by surveying cytokine and microglia findings in the most widely used animal model of ELS. RMS is considered to have etiological validity as an analogue of human ELS because it occurs in a period of early life analogous to early childhood in humans, and its effects are mediated by reduced parental attendance to emotional and physical needs, just as they are in caregiver neglect, the most prevalent form of human childhood maltreatment (Schmidt et al., 2011; Semple et al., 2013).

**Figure 3.**
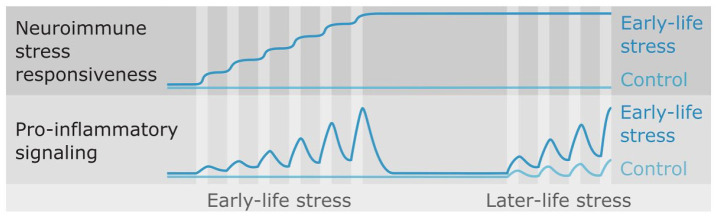
Hypothesised effects of early-life stress (ELS). The findings of this review suggest that ELS exerts a long-lasting augmentation to individuals’ physiological responsiveness to stressors. When exposed to stressors later in life, individuals with a history of ELS may exhibit elevated autonomic nervous or endocrine signalling, and/or elevated immune cell responses to that signalling, and in turn elevated pro-inflammatory cytokine expression.

Overall, RMS did not appear to cause a persistent production of a pro-inflammatory state by immune cells, given that among included studies, where animals experienced no later-life stress, generally no long-term effect of RMS on cytokine expression was found. However, in the context of later-life stress, RMS animals very commonly exhibited a more pro-inflammatory cytokine expression profile than controls. The contrast between these two sets of findings suggests that RMS causes a long-lasting sensitization of the mechanism by which an active psychosocial stressor results in pro-inflammatory signalling in tissues (summarised in Figure 3). In this mechanism as presently understood, (1) the CNS makes an assessment of stressor intensity and generates a proportional systemic production of neurotransmitters and hormones such as noradrenaline, adrenaline, and cortisol, which then act directly and indirectly on (2) innate immune cells, which respond by altering their production of pro-inflammatory cytokines (Fleshner and Crane, 2017; Miller and Raison, 2016; Ulrich-Lai and Herman, 2009; Weber et al., 2017). It is possible that the sensitising effects of RMS are mediated through modification of either or both components of this mechanism. To our knowledge, only one study has been conducted to date that directly assessed these two possibilities. Kiank et al. (2009) harvested splenocytes, a rich collection of innate and adaptive immune cells, from RMS and control rats and performed an ex vivo assessment of their cytokine production in response to LPS, avoiding the confounding of the stress caused by in vivo LPS administration. They found differences in cytokine production by immune cells between RMS and control animals only when both groups experienced later-life stress; in the absence of later-life stress, LPS-stimulated cytokine production by immune cells was completely unaffected by RMS. This suggests that rather than priming innate immune cells to effect a greater pro-inflammatory response to activating signals, RMS may instead result in greater nervous and/or neuroendocrine production of activating signals in response to later-life stress. Indeed, many studies in both humans and animals have reported long-lasting effects of ELS on the brain which could result in increased autonomic and endocrine responsiveness to stress. For example, RMS has been reported to increase neuron density in the amygdala (Bassey and Gondré-Lewis, 2019; Gondré-Lewis et al., 2016) and decrease parvalbumin-positive interneuron density in the medial prefrontal cortex (mPFC) (Do Prado et al., 2016; Grassi-Oliveira et al., 2016; Wieck et al., 2013), while human ELS has been shown to decrease dorsal mPFC volume in adulthood (Van Harmelen et al., 2010). However, in rodent models of adulthood stress, stress-induced priming of immune cells has been demonstrated both to subsequent stressors (Audet et al., 2011) and inflammatory stimuli (Frank et al., 2014; Wohleb et al., 2012), indicating that further work is required to understand the role of immunological priming in the elevated neuroimmune responsiveness that follows ELS.

Another implication of our findings is that the elevated inflammation that has been identified in humans with a history of ELS may be a direct result of (1) ongoing adulthood stress, on a background of (2) increased responsiveness to stress. Ongoing adulthood stress may, for example, result from everyday occupational, financial, or relationship stressors, or from the consequences of one or more of the psychiatric disorders that individuals with a history of ELS are at increased risk of developing. It is also possible that individuals with a history of ELS exhibit a more intense stress–immune response to sample collection itself, in which case findings suggesting elevated inflammation should be interpreted not necessarily as reflecting a stable elevated baseline, but instead as suggesting increased variability and peak daily pro-inflammatory signalling or increased average inflammation over time. These findings point towards opportunities for therapeutic intervention in patients with a history of ELS, aimed at preventing or treating disorders thought to be caused or exacerbated by inflammation, such as major depression and cardiovascular disease (Batten et al., 2004; Nanni et al., 2012; Steptoe and Kivimäki, 2012). Identification and reduction of ongoing stressors, as well as certain psychotherapeutic, meditation, and relaxation regimens, represent readily available non-pharmacological interventions that, given our findings, may be particularly beneficial for patients with a history of ELS (Antoni et al., 2012; Creswell et al., 2012; Morgan et al., 2014; Pace et al., 2009).

While we did not collect data directly examining the causal role of ELS-associated inflammation with respect to any particular disorders, our findings have some generalizable implications regarding causality. Where long-term non-immunological effects of RMS have been identified in animals not subjected to any further stress, such as neurobiological effects (Matthews et al., 2001), our results suggest that ongoing pro-inflammatory signalling may be unlikely to play a causal role in those effects. However, the immune system may still be causally involved in that the early-life inflammation may have long-lasting effects on other systems or processes, for example, neurodevelopment, which persist beyond the resolution of the early-life inflammation (Estes and McAllister, 2016; Knuesel et al., 2014). Intervention studies targeting the immune system, particularly in early life, are necessary to elucidate the precise consequences of RMS-associated inflammation, and this review provides clear guidance for such studies. In RMS animals, it should be expected that pro-inflammatory signalling will peak during or immediately after early-life or later-life stress exposure and rapidly decline in the absence of stress. Normalisation of inflammatory signalling should be expected within several weeks if not within 1 day (Roque et al., 2016), although recovery may be slowed if animals are deprived of standard stress-relieving cage elements such as tunnels, nesting, and littermates (Do Prado et al., 2016). Therefore, both inflammation and its hypothesised consequences should be measured during or immediately after stress, and interventions targeting the immune system will likely be most effective if administered during early-life or later-life stress, or both. In addition, measurement of inflammatory signalling in non-blood tissues relevant to disorders of interest is encouraged, as this appears to be more sensitive than measurement in blood. For three out of four cytokines, the most common short-term effect in non-blood tissues was an increase, whereas in the blood, no change was most common. In addition, while most included studies measured cytokine expression in either non-blood tissue or blood, but not both, four studies did simultaneously measure a particular cytokine in both types of tissue, and three of these detected at least one change in non-blood tissue that was not detected in plasma (Moya-Pérez et al., 2017; Roque et al., 2016; Wang et al., 2017).

In summary, our findings in animals subjected to ELS suggest that ELS results in a long-lasting sensitization of the neuroimmune response to stress, and consequently a propensity to elevated inflammation in response to later-life stress. Furthermore, the finding that for ELS to result in elevated inflammation in later life, subjects must generally be exposed to ongoing stress, suggests that non-pharmacological interventions aimed at reducing stressor exposure or stress responsiveness may be particularly beneficial in reducing inflammation in people with a history of ELS. Finally, our findings guide the future use of RMS to interrogate the causal roles of ELS and inflammation in disorders such as depression and cardiovascular disease.

## Supplemental Material

supplementary_information – Supplemental material for Early-life stress and inflammation: A systematic review of a key experimental approach in rodents Click here for additional data file. Supplemental material, supplementary_information for Early-life stress and inflammation: A systematic review of a key experimental approach in rodents by Ethan G. Dutcher, E.A. Claudia Pama, Mary-Ellen Lynall, Shahid Khan, Menna R. Clatworthy, Trevor W. Robbins, Edward T. Bullmore and Jeffrey W. Dalley in Brain and Neuroscience Advances
