# Supplementary material for: Early-life stress and inflammation: A systematic review of a key experimental approach in rodents
Source: Brain Neurosci Adv. 2020 Dec 28;4:2398212820978049. doi: 10.1177/2398212820978049 (PMC7780197; doi:10.1177/2398212820978049)
Supplement: supplementary_information – Supplemental material for Early-life stress and inflammation: A systematic review of a key experimental approach in rodents [file supplementary_information.pdf]

Supplementary Methods

A search was conducted of the PubMed and Embase databases via Scopus to identify publications for which the title, abstract, and keywords together contained any of the terms "maternal separation", "early deprivation", or "maternal deprivation", with at least one of the following additional terms: cytokine*, chemokine*, interleukin*, microglia*, monocyt*, "immune", "immunological", "immunity", "neuro-immunological", "mononuclear", "toll-like", and "toll like". Publications were limited to those classified as articles. The search was performed on 18 December 2019 and results were not restricted by date range. Articles were included if they performed either single-episode or repeated maternal separation (MS) in rats or mice and specifically reported on measures of microglial activation or density, or the expression of IL-1β, TNF-α, IL-6, or IL-10 in any tissue. Measurements were classified as reflecting short-term effects of MS if the measurement was taken within three weeks of the conclusion of MS, or as reflecting long-term effects if they were taken over three weeks after MS concluded. Three weeks was selected as the cut-off because many disruptions to behavioral and non-immune physiological parameters normalize by this time following chronic stress in rodents, but not reliably by earlier times (Alves et al., 2017; Jacobson et al., 2018; Park et al., 2017; Zhao et al., 2012). Short-term measurements were excluded from this review if they were taken from animals subjected to any potentially stressful procedure other than MS, except for intraperitoneal saline injection. Findings were excluded if the approximate age of the animals at the time of measurement was unclear (general descriptions such as "adulthood" were acceptable) or if potentially therapeutic interventions were administered to animals, and articles were excluded if the full text was inaccessible. We did not restrict our search to particular tissues, because nervous-to-immune communication is thought to occur via altered autonomic nervous and neuro-

endocrine signaling (Fleshner and Crane, 2017; Miller and Raison, 2016; Weber et al., 2017), and stress-induced effects on these pathways are known to occur concurrently in numerous organ systems and tissues throughout the body (Lupien et al., 2009; Ulrich-Lai and Herman, 2009). The search yielded 220 results, which were screened against the inclusion and exclusion criteria by the reading of abstracts and full text as appropriate. Ultimately, 46 articles met criteria for inclusion.

The findings of included articles were described in two ways. A written synthesis of the articles was created, and reference was made in writing to every finding that met the above criteria. The written descriptions and summaries were organized according to whether the findings measured short-term or long-term effects as defined above, and in the latter case, whether or not animals experienced stressful conditions or procedures following the conclusion of RMS. A semi-quantitative summary table was then created, containing the most commonly reported outcome (increase, decrease, or no change) for each cytokine under each time-stress condition: short-term without stress, long-term without stress, and long-term with stress. Microglial outcomes were not counted due to the low number of applicable studies. Because several studies reported a discrepancy between the short-term effect of RMS on cytokine expression in the blood compared to in a non-blood tissue (Moya-Pérez et al., 2017; Roque et al., 2016; Wang et al., 2017), measures in blood (plasma, serum, or supernatant of cultured whole blood) and non-blood tissue were treated separately. For each cytokine under each condition, each study's findings were reduced to a single summary finding for non-blood tissue and a single finding for blood. Where a study reported an applicable measurement and found an increase or decrease in expression in at least one non-blood tissue or blood product, that study was counted as supporting either an increase or decrease respectively in that particular tissue class, and otherwise the study was

considered to support no change. Where a study reported the same measurement at two or more times within the same time-stress condition, only the first measurement was counted. No within-study conflicts were encountered using this process. The single most common outcome was reported for each combination of cytokine, time-stress condition, and tissue type, except where there was a tie or where two outcomes had the support of at least three studies, in which case both outcomes were reported. Where outcomes had the support of at least three studies, this was indicated as a measure of higher confidence in the summary outcome.

Supplementary Figures

		Short-term		Long-term without stress		Long-term with stress	
		Protein	mRNA	Protein	mRNA	Protein	mRNA
Non-Blood	IL-1β	↔	↓	↔	↔	↑	↑
	TNF-α	↑	↑	↔	↔	↑ / ↔	↑
	IL-6	↑	↑	↔	↔	↑	↑
	IL-10	↑	↓	↔	↔	↓	↑ / ↓

Figure S1. **Effects of repeated maternal separation (RMS) on cytokine expression in non-blood tissue, segregated by messenger RNA (mRNA) and protein.** The most commonly reported outcomes (increase, decrease, or no change) are summarized for each cytokine for each stress condition, counting mRNA and protein results separately. Dark blue shading indicates a high level of confidence (three or more studies supporting each outcome), whereas light blue shading indicates low confidence.

Study	Species	Gender	Non-blood tissue assay substrate	Later-life stress
**Amini-Khoei 2017**	Mouse	Male	mRNA	Forced swim test and open field test
**Amini-Khoei 2019**	Mouse	Male	mRNA	Forced swim test, open field test, and elevated plus maze
**Avitsur 2006**	Mouse	Both	Protein (IL-6), mRNA (IL-1b, TNF-a, IL-6)	Influenza virus infection (inoculation nine days prior to sacrifice)
**Avitsur 2013**	Mouse	Both	N/A	Isolation housing for one day then a single IP injection of either saline, low-dose LPS, or high-dose LPS two hours before sacrifice
**Baldy 2018**	Rat	Both	N/A	N/A
**Banqueri 2019**	Rat	Male	mRNA	N/A
**Barouei 2015**	Rat	Both	N/A	Four days of thirty minutes daily restraint or isolation stress, immediately before sacrifice
**Barreau 2004**	Rat	Male	mRNA	N/A
**Breivik 2015**	Rat	Both	N/A	Three weeks of ligature-induced periodontitis and then an LPS injection two hours before sacrifice

**Carboni 2010**	Rat	Male	N/A	Forced swim test roughly a week before sacrifice in depression-prone rats
**Dalle 2016**	Rat	Male	mRNA	Intracerebral injection of the neurotoxin 6-hydroxydopamine to lesion the dopaminergic projections passing through the medial forebrain bundle
**De Miguel 2018**	Rat	Male	Protein	IP injection of either saline or low-dose LPS fourteen hours before sacrifice
**Desbonnet 2010**	Rat	Male	N/A	Forced swim test
**Dimatelis 2012**	Rat	Male	mRNA	N/A
**Do Prado 2016**	Rat	Both	N/A	Lack of enrichment other than one cage-mate for five weeks between weaning and sacrifice
**Fuentes 2016**	Mouse	Male	mRNA	One-hour water avoidance stress
**Ganguly 2018**	Rat	Both	N/A	N/A
**Ganguly 2019**	Rat	Both	Protein (TNF-a), mRNA (TNF-a)	IP injection of saline four hours before sacrifice
**Genty 2018**	Rat	Male	mRNA	Compression trauma to a peripheral nerve
**Giridharan 2019**	Rat	Male	Protein	N/A

**Grassi-Oliveira 2016**	Rat	Both	N/A	N/A
**Han 2019**	Mouse	Male	Protein (IL-6), mRNA (IL-1b, TNF-a, IL-6)	Two weeks of IP saline injections followed in some animals by two weeks of two hours daily restraint
**Kiank 2009**	Mouse	Female	Protein	Five days of restraint and acoustic noise stress for two to four hours daily
**Kruschinski 2008**	Rat	Male	mRNA	Sensitization to ovalbumin by IP injection one and two weeks before sacrifice then tracheal instillation of ovalbumin a day before sacrifice
**Lennon 2013**	Mouse	Unclear	mRNA	N/A
**Li 2017**	Mouse	Unclear	mRNA	N/A
**Majcher-Maslanka 2019**	Rat	Both	mRNA	N/A
**Mizoguchi 2019**	Mouse	Male	N/A	Compression trauma to a peripheral nerve
**Moya-Perez 2017**	Mouse	Male	Protein	N/A
**O'Mahony 2009**	Rat	Male	N/A	Repeated brief open field testing
**O'Malley 2011**	Rat	Male	Protein	N/A
**Pierce 2014**	Mouse	Female	mRNA	Abdominal wall surgery and visceral organ distension to assess pain sensitivity, a week prior to sacrifice

**Pierce 2016**	Mouse	Female	mRNA	One-hour water avoidance stress and either sacrifice next day or sacrifice eight days later with three tail vein bleeds during that time
**Pinheiro 2014**	Rat	Male	Protein	Two weeks of daily IP saline injections then brief behavioral testing including open field test
**Reus 2013**	Rat	Male	Protein	Two weeks of daily IP saline injections with forced swim testing on the final two days, with sacrifice immediately after the final test
**Riba 2017**	Mouse	Unclear	Protein	N/A
**Riba 2018**	Mouse	Male	Protein	N/A
**Roque 2016**	Rat	Male	mRNA	N/A
**Saavedra 2017**	Rat	Male	Protein	N/A
**Shao 2019**	Rat	Male	mRNA	Abdominal wall electrode implantation five days prior to sacrifice and then testing for sensitivity to organ distension
**Tang 2017**	Mouse	Unclear	Protein	Colorectal distension for visual pain sensitivity scoring

**Vig 2010**	Mouse	Both	Protein	IP injection of ovalbumin nine and fourteen days prior to sacrifice, then intranasal inoculation of ovalbumin or saline one and three days prior to sacrifice
**Viola 2019**	Mouse	Male	mRNA	IP injection of poly-(I:C) five hours prior to sacrifice
**Wang 2017**	Mouse	Male	Protein	Three weeks of daily IP saline injections ending two weeks before sacrifice, followed by behavioral tests including elevated plus maze and open field test
**Zajdel 2019**	Mouse	Both	mRNA	N/A
**Zhu 2017**	Rat	Male	Protein	Two-hour sevoflurane anesthetic three to five days prior to sacrifice

Figure S2. **Study characteristics.** The gender, species, and assay substrate are listed for each included study. For those included studies in which repeated maternal separation (RMS) was conducted followed by a procedure in late adolescence or adulthood that was likely to be stressful, the relevant procedure(s) applied in those studies are listed.

Study	Follow-up type	Microglial activation	Microglial density
**Baldy 2018**	Short-term	↑ (medulla)	↑ (medulla)
**Majcher-Maślanka 2019**	Short-term	N/A	↓ (prelimbic PFC)
**Roque 2016**	Short-term	↑ (hippocampus)	↔ (hippocampus)
**Saavedra 2017**	Short-term	↑ (hippocampus)	↓ (hippocampus)
**Banqueri 2019**	Long-term without stress	N/A	↑ (hippocampus, dorsal striatum, nucleus accumbens)
**Ganguly 2018**	Long-term without stress	↔ (prelimbic PFC)	↔ (prelimbic PFC)
**Han 2019**	Long-term with stress	↑ (hippocampus)	N/A
**Mizoguchi 2019**	Long-term with stress	N/A	↑ (spinal cord)

Figure S3. **Microglial outcomes.** The results of included studies measuring the effect of RMS on microglia activation or density are listed, as increase, decrease, or no change.

References

Alves ND, Correia JS, Patrício P, et al. (2017) Adult hippocampal neuroplasticity triggers susceptibility to recurrent depression. *Translational psychiatry* 7(3): e1058.

Fleshner M and Crane CR (2017) Exosomes, DAMPs and miRNA: Features of Stress Physiology and Immune Homeostasis. *Trends in immunology* 38(10): 768–776.

Jacobson ML, Kim LA, Patro R, et al. (2018) Common and differential transcriptional responses to different models of traumatic stress exposure in rats. *Translational psychiatry* 8(1): 1–12.

Lupien SJ, McEwen BS, Gunnar MR, et al. (2009) Effects of stress throughout the lifespan on the brain, behaviour and cognition. *Nature reviews. Neuroscience* 10(6): 434–445.

Miller AH and Raison CL (2016) The role of inflammation in depression: from evolutionary imperative to modern treatment target. *Nature reviews. Immunology* 16(1): 22–34.

Moya-Pérez A, Perez-Villalba A, Benítez-Páez A, et al. (2017) Bifidobacterium CECT 7765 modulates early stress-induced immune, neuroendocrine and behavioral alterations in mice. *Brain, behavior, and immunity* 65: 43–56.

Park SE, Park D, Song K-I, et al. (2017) Differential heart rate variability and physiological responses associated with accumulated short- and long-term stress in rodents. *Physiology & behavior* 171: 21–31.

Roque A, Ochoa-Zarzosa A and Torner L (2016) Maternal separation activates microglial cells and induces an inflammatory response in the hippocampus of male rat pups, independently of hypothalamic and peripheral cytokine levels. *Brain, behavior, and immunity* 55: 39–48.

Ulrich-Lai YM and Herman JP (2009) Neural regulation of endocrine and autonomic stress responses. *Nature reviews. Neuroscience* 10(6): 397–409.

Wang Q, Dong X, Wang Y, et al. (2017) Adolescent escitalopram prevents the effects of maternal separation on depression- and anxiety-like behaviours and regulates the levels of inflammatory cytokines in adult male mice. *International journal of developmental neuroscience the official journal of the International Society for Developmental Neuroscience* 62: 37–45.

Weber MD, Godbout JP and Sheridan JF (2017) Repeated Social Defeat, Neuroinflammation, and Behavior: Monocytes Carry the Signal. *Neuropsychopharmacology official publication of the American College of Neuropsychopharmacology* 42(1): 46–61.

Zhao Y, Wang Z, Dai J, et al. (2012) Beneficial effects of benzodiazepine diazepam on chronic stress-induced impairment of hippocampal structural plasticity and depression-like behavior in mice. *Behavioural brain research* 228(2): 339–350.
